# Ex vivo anchored PD‐L1 functionally prevent in vivo renal allograft rejection

**DOI:** 10.1002/btm2.10316

**Published:** 2022-04-06

**Authors:** Zihuan Luo, Tao Liao, Yannan Zhang, Haofeng Zheng, Qipeng Sun, Fei Han, Maolin Ma, Yongrong Ye, Qiquan Sun

**Affiliations:** ^1^ Department of Renal Transplantation Guangdong Provincial People's Hospital, Guangdong Academy of Medical Sciences Guangzhou Guangdong China; ^2^ The Second School of Clinical Medicine Southern Medical University Guangzhou Guangdong China; ^3^ Organ Transplantation Research Institute The Third Affiliated Hospital of Sun Yat‐sen University Guangzhou Guangdong China

**Keywords:** cell surface engineering, induction therapy, PD‐L1, rejection, transplantation

## Abstract

Organ transplantation is the optimal treatment for patients with end‐stage diseases. T cell activation is a major contributing factor toward the trigger of rejection. Induction therapy with T cell depleting agent is a common option but increases the risk of severe systemic infections. The ideal therapy should precisely target the allograft. Here, we developed a membrane‐anchored‐protein PD‐L1 (map‐PD‐L1), which effectively anchored onto the surface of rat glomerular endothelial cells (rgEC). The expression of PD‐L1 increased directly with map‐PD‐L1 concentration and incubation time. Moreover, map‐PD‐L1 was even stably anchored to rgEC at low temperature. Map‐PD‐L1 could bind to PD‐1 and significantly promote T cell apoptosis and inhibited T cell activation. Using kidney transplantation models, we found that ex vivo perfusion of donor kidneys with map‐PD‐L1 significantly protected grafts against acute injury without using any immunosuppressant. We found map‐PD‐L1 could reduce T cell graft infiltration and increase intragraft Treg infiltration, suggesting a long‐term effect in allograft protection. More importantly, modifying donor organs in vitro was not only safe, but also significantly reduced the side effects of systemic application. Our results suggested that ex vivo perfusion of donor organ with map‐PD‐L1 might provide a viable clinical option for organ‐targeted induction therapy in organ transplantation.

## 1 INTRODUCTION

Organ transplantation can be the final solution for patients suffering end‐stage organ disease.^1^ The recipient's immune system is exposed to a large amount of alloantigens during transplantation, usually leading to rejection during surgery.^2^ Because the alloreactive immune response is initiated at the interface between the graft endothelium and the recipient's T cells,^3^ nearly 75% of recipients receive induction therapy with T cell depleting agents.^4^ However, these approaches have been found to be less beneficial than initially expected due to the occurrence of adverse systemic effects, including the development of severe bacterial, viral, and fungal infections.^5^, ^6^ Research into new immunosuppressive agents that are able to precisely target the allograft and hence, avoid systemic effects, is urgently needed to improve the prognosis of transplant patients while inducing minimal side effects.

Programmed cell death protein‐1 (PD‐1) and its ligand PD‐L1 are important immune checkpoint molecules that negatively regulate the immune system.^7^, ^8^ It has been reported that PD‐L1 represses rejection in transplantation, and lack of PD‐L1 has been shown to reduce survival time after islet transplantation.^9^ In recent years, many research groups have attempted to upregulate PD‐L1 expression in grafts through viral transfection and genetic engineering, which has produced a positive effect on the survival of grafts.^10^, ^11^ However, these methods are unable to achieve selective targeting, and the risk of systemic administration still hinders their further clinical application. Therefore, a fast, efficient and simple method to upregulate PD‐L1 expression in organs will promote the application of PD‐L1‐based therapy in transplantation immunity.

Cell surface engineering is an effective technique for expressing new proteins on cell membranes without gene transfer. The key component of this technique is the membrane anchoring structure, which secures the target protein to the surface of the cell.^12^, ^13^ Through this, the target protein can be “implanted” on the cell surface, and the innate biological activity and function of the protein can be preserved.^14^ Using this principle, we have successfully constructed membrane‐anchored‐protein PD‐L1 (map‐PD‐L1), a fusion protein consisting of PD‐L1 and APT542 (a membrane‐anchored protein structure). One useful feature of organ transplantation is the ex vivo accessibility of the organ, as extracorporeal treatment of donor organs can effectively avoid systemic side effects. However, it is unclear whether map‐PD‐L1 can anchor to the donor organ in vitro and ultimately lead to high expression of PD‐L1 in donor organs.

Here, we verified the biological function and anchoring ability of map‐PD‐L1 using in vitro experiments, and tested the hypothesis that preperfusion of the donor kidney with map‐PD‐L1 prior to kidney transplantation could reduce early intragraft inflammation and prevent acute rejection.

## 2 MATERIAL AND METHODS

### 2.1 Design and synthesis of the map‐PD‐L1

The protein amino acid sequence of rat‐derived PD‐LI and anchoring structure APT542 were codon optimized using MaxCodonTM Optimization Program (V13) (DetaiBio). The PD‐L1 + APT542 gene was inserted into the expression vector pcDNA3.1(−) by whole‐genome synthesis and double enzyme digestion. Accuracy of the final expression vector was confirmed by restriction enzyme digestion and sequencing. Finally, the vector was transformed into DH5a cells, and the plasmid was extracted using a plasmid extraction kit. The plasmid was then transfected into mammalian HEK293 cells for transient expression, and the fusion protein map‐PD‐L1 was purified using affinity chromatography. The expression and specificity of the synthesized protein was verified by Western blot. The membrane‐localizing thiol‐reactive agent APT542 was generated by solid‐phase synthesis, and APT542 without PD‐L1 was used as a control in this study.

### 2.2 Cell line

Rat glomerular endothelial cells (rgEC) were kindly provided by Hui Peng (The Third Affiliated Hospital of Sun Yat‐sen University). rgEC were incubated in RPMI‐1640 supplemented with 10% FBS and 10% NuSerum (Sigma‐Aldrich) in a cell incubator at 37°C under 5% CO_2_, as previously described.^15^

### 2.3 Immunofluorescence

To verify the ability of map‐PD‐L1 to anchor to the cell membrane, we incubated rgEC with 5 μg/ml map‐PD‐L1 or PD‐L1 at 37°C for 1 h. Cells were then fixed in 4% paraformaldehyde (C01‐06002; Bioss), permeabilized with 0.1% Triton X‐100 (C03‐03001; Bioss) in phosphate‐buffered saline (PBS), and incubated with rabbit anti‐PD‐L1 polyclonal antibody (20 μg/ml; NBP1‐76769; Novus Biologicals) overnight at 4°C in humidified chambers. The cells were then incubated with Alexa Fluor‐conjugated secondary antibodies (1:100; CA11012S; Invitrogen) for 1 h at room temperature. Sections were visualized with 4′‐6′‐diamidino‐2‐phenylindole (DAPI; Sigma‐Aldrich) and then examined using confocal laser scanning microscope (Zeiss LSM880).

### 2.4 Flow cytometry

To explore the efficiency of map‐PD‐L1 anchoring to rgEC, a cell suspension was generated, consisting of 1 × 10^5^ per tube. Subsequently, cells were washed twice with PBS and then incubated with fluorescein isothiocyanate (FITC)‐labeled map‐PD‐L1 at different concentrations, temperatures, and incubation times. The cells were then fixed in 4% paraformaldehyde (C01‐06002; Bioss) and analyzed using a FACscan flow cytometer (Becton Dickinson).

### 2.5 Coculture test

Referring to the above method, rgEC were cultured in a 24‐well cell culture plate (Corning Inc.). When the cell density reached at least 50%, 50 μg/ml of map‐PD‐L1 were added to the experimental group and incubated for 30 min at 37°C. For the control group, an equal volume of medium was added instead. After incubation, cells were washed twice with PBS, and then preacquired Lewis rat spleen lymphocytes were cocultured with the rgEC at a density of 2 × 10^6^ cells/well.

For the T cell apoptosis assay, cells were cocultured for 3 days. The supernatant was centrifuged to obtain mixed cultured lymphocytes, which were then washed twice with PBS and incubated with APC anti‐rat CD3 (1 μg/ml; 130‐103‐132; Miltenyi Biotec) and FITC Annexin V Apoptosis Detection Kit I (1 μg/ml; 556547; BD Biosciences). T cell apoptosis was detected by flow cytometer (Becton Dickinson).

For the T cell activation assay, cells were cocultured with anti‐CD3 (1 μg/ml; ab16669 Abcam) and anti‐CD28 (1 μg/ml; ab243228; Abcam) antibodies for 1 day as a stimulation protocol. Then the supernatant was centrifuged to obtain mixed cultured lymphocytes, which were then washed twice with PBS and incubated with APC anti‐rat CD3 (1:100; 130‐103‐132; Miltenyi Biotec) and PE‐Cy7 anti‐rat CD69 (1:100; bs‐2499R; Bioss) antibodies. T cell activation was detected by flow cytometer (Becton Dickinson).

To analyze apoptosis of rgEC, cells were cocultured with anti‐CD3 (1 μg/ml; ab16669; Abcam) and anti‐CD28 (1 μg/ml; ab243228; Abcam) antibodies for 3 days as a stimulation protocol. After the supernatant was removed, the remaining adherent cells were washed twice with PBS and detected using a TUNEL Apoptosis Assay Kit (C1086; Beyotime).

### 2.6 Kidney transplantation and treatment with map‐PD‐L1

Adult male Lewis rats and Brown Norway (BN) rats weighing 225–250 g were obtained from Charles River Laboratories (Beijing, China) and bred in a pathogen‐free room at Sun Yat‐sen University. All animal experiments were performed in accordance with the Guide for the Care and Use of Laboratory Animals (National Institutes of Health publication No. 80‐23, revised 1996) and according to the Sun Yat‐sen University Institutional Ethical Guidelines for animal experiments.

The rats were divided into the following groups: map‐PD‐L1 (anchored map‐PD‐L1), APT542 (APT542 anchoring) and control (simple perfusion solution). Donor kidney procurement and transplantation were performed under anesthesia with isoflurane as described previously.^16^ Briefly, the left donor kidney of the BN rat was obtained, then perfused with 5 ml of UW (University of Wisconsin) perfusion solution containing 200 μg of map‐PD‐L1 over a 5‐min period. The kidney was then placed in ice‐cold UW solution for 30 min, and reperfused to remove any unbound map‐PD‐L1 before transplantation. The recipient Lewis rat was anesthetized. The aorta, renal vein, and ureter of the graft were anastomosed to the aorta, inferior vena cava, and ureter, respectively, of the recipient using 8‐0 sutures. Both native kidneys were excised at the time of engraftment, and the total surgical ischemia time was restricted to less than 45 min. The control and blank groups were also treated by the process detailed above.

### 2.7 Graft harvesting

To evaluate the transplanted kidney function of both the experimental and control groups, blood samples were collected from the groups before surgery, and 1, 3, and 5 days after surgery. The samples were centrifuged, and stored at a low temperature. Serum creatinine and blood urea nitrogen (BUN) levels were measured using the appropriate kits. During the follow‐up, the survival time was recorded.

### 2.8 Histologic analysis of allograft rejection

To assess the pathological changes and inflammatory cell infiltration of the transplanted kidney, the graft was obtained 5 days after transplantation, then fixed with 4% paraformaldehyde and embedded with paraffin. Histologic sections for light microscopy were cut to a thickness of 3 μm, stained with hematoxylin and eosin (H&E) as well as Periodic acid–Schiff (PAS), and reviewed by an experienced pathologist who was blinded to the groups. Cell‐mediated injuries included interstitial inflammation, tubulitis and acute tubular necrosis were scored by the semiquantitative Banff scoring criteria: 0, absent; 1, mild; 2, moderate; 3, prominent.^17^, ^18^

### 2.9 Immunohistochemistry and immunofluorescence

For immunohistochemistry analysis, 3 μm thick cross‐sections were deparaffinized and rehydrated, and then incubated at 4°C overnight with the following primary antibodies: anti‐CD3 (1:800; ab33429; Abcam), anti‐CD4 (1:800; ab203034; Abcam), anti‐CD8 (1:1000; ab217344; Abcam), anti‐CD68 (1:600; ab125212; Abcam), and anti‐FOXP3 (1:800; ab215206; Abcam). Then, the samples were stained with goat anti‐rabbit IgG/HRP (1:100; bs‐0295G‐HRP; Bioss) for 1 h at 37°C. Semi‐quantitative analysis of the inflammation characteristics in samples was performed using Image‐Pro Plus (IPP) 6.0 imaging software (Media Cybernetics). Immunofluorescence was used to detect the expression of PD‐L1 (20 μg/ml; NBP1‐76769; Novus Biologicals) in the grafts and the samples were examined using confocal laser scanning microscope (Zeiss LSM880).

### 2.10 Quantitative real‐time polymerase chain reaction

Total RNA was extracted from frozen kidney tissue using TRIzol reagent (Invitrogen) on homogenized samples. A total of 2 μg of RNA was reversed‐transcribed using PrimeScript RT Master Mix (Perfect Real Time) (TAKARA). Transcript‐specific primers were designed using Primer Express software and verified using NCBI Primer Blast. The sequences of the primer pairs are listed in Table S2. Real‐time reverse‐transcription polymerase chain reaction (RT‐PCR) was performed in triplicate using LightCycler 480 SYBR Green I Master (Roche) in a Roche LightCycler480 System. For each gene, primer pairs generated a single product and normalized to the housekeeping *GAPDH* gene. The2^−△△*CT*^ method was used for calculation.

### 2.11 Enzyme‐linked immunosorbent assay

To detect the binding between the recombinant map‐PD‐L1 and PD‐1 we used the enzyme‐linked immunosorbent assay (ELISA)‐based assay. We redesigned an in vitro experiment and divided the experiment into two groups: the experimental group used 50 μg/ml map‐PD‐L1 to pretreat endothelial cells, while the control group used 50 μg/ml His‐tagged PD‐L1 (80450‐R08H; SinoBiological) recombinant protein; different concentration gradients of hFc‐tagged PD‐1 (80448‐R02H; SinoBiological) recombinant protein (0.01 ng/ml, 0.1 ng/ml, 1 ng/ml, 10 ng/ml,100 ng/ml, 1 μg/ml) were added to the wells and incubated for 1 h at 37°C and then washed three times in ELISA wash buffer. Then, a horseradish peroxidase (HRP)‐conjugated anti‐Human IgG‐Fc (anti‐hFc) (SSA001; SinoBiological) was added to the wells (0.1 μg/ml) and incubated at 37°C for 1 h and then washed three times in ELISA wash buffer. HRP activity was detected by using ELISA TMB Stabilized Chromogen (SB01; Thermo Fisher Scientific) following the manufacturer's instructions.

Blood samples from recipients at different time points were used to determine the serum concentration of PD‐L1 using the ELISA according to the manufacturer's instruction (MBS070715; MyBioSource).

### 2.12 Body weight and feed utilization

The recipients were observed daily, and body weight and feed consumption were recorded interval day. Feed utilization was determined using the following calculation: Feed utilization (%) = (body weight gain/feed consumption) × 100.

### 2.13 Statistical analysis

Normal distribution was first used to test the distribution of data using KS normality test. All data were normal distribution. Differences between groups were evaluated using either Student's *t*‐test or analysis of variance using SPSS 19.0 software (SPSS Inc.). Graft survival among groups was compared using a log rank test. Data are shown as mean ± SD. A *p* < 0.05 was considered statistically significant.

## 3 RESULTS

### 3.1 Study design and synthesis process and identification of map‐PD‐L1

**FIGURE 1:**
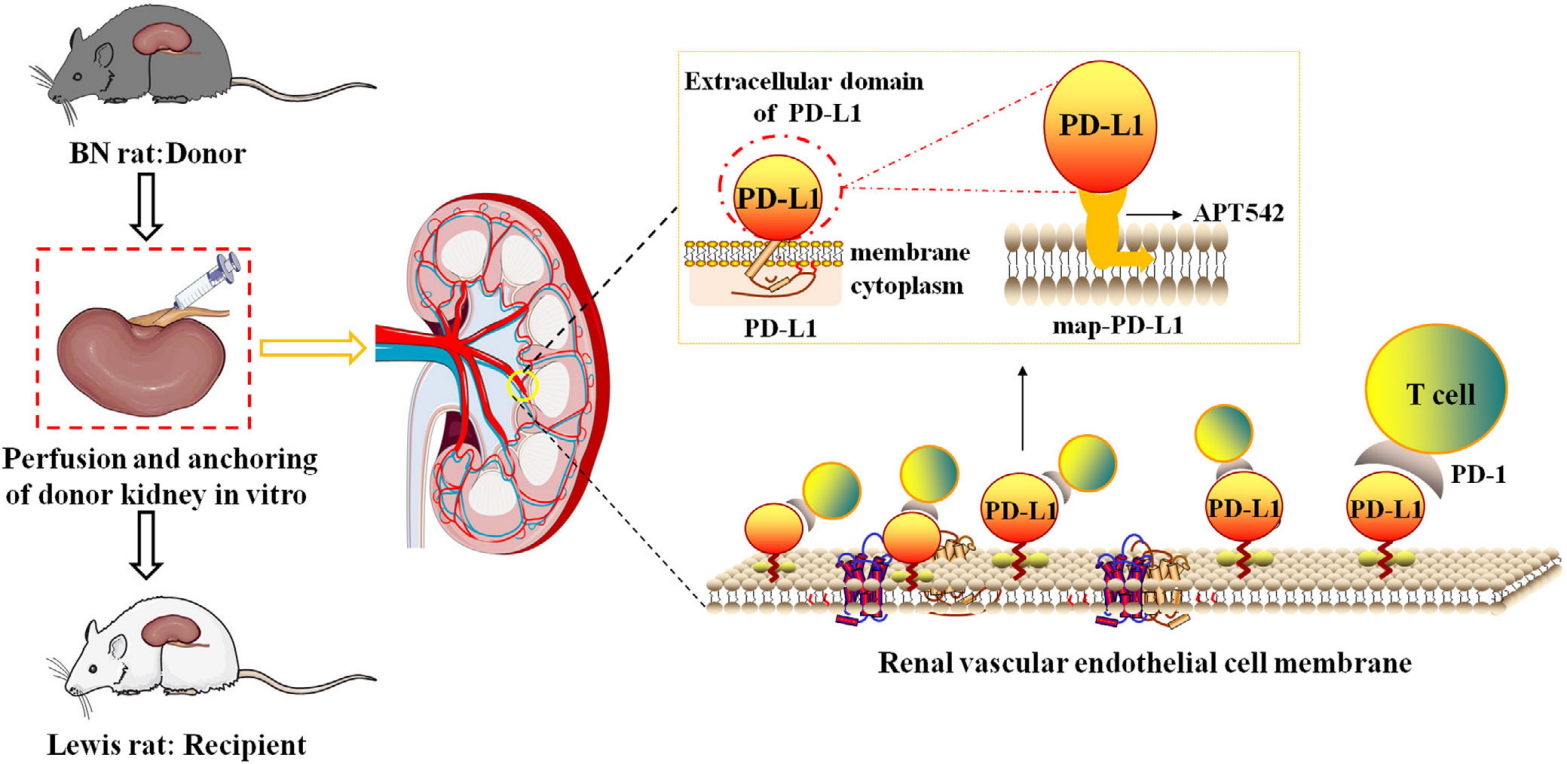
Study design. The recombinant protein map‐PD‐L1, which retains the function of PD‐L1 and is anchored to the cell membrane, was expressed (see the yellow dashed box schematic). The ability to perform ex vivo perfusion is an important feature of the kidney. The kidneys were treated with a perfusion solution premixed with map‐PD‐L1, and map‐PD‐L1 was “implanted” on the surface of renal endothelial cells by incubation for the appropriate time. map‐PD‐L1, membrane‐anchored‐protein PD‐L1

**FIGURE 2:**
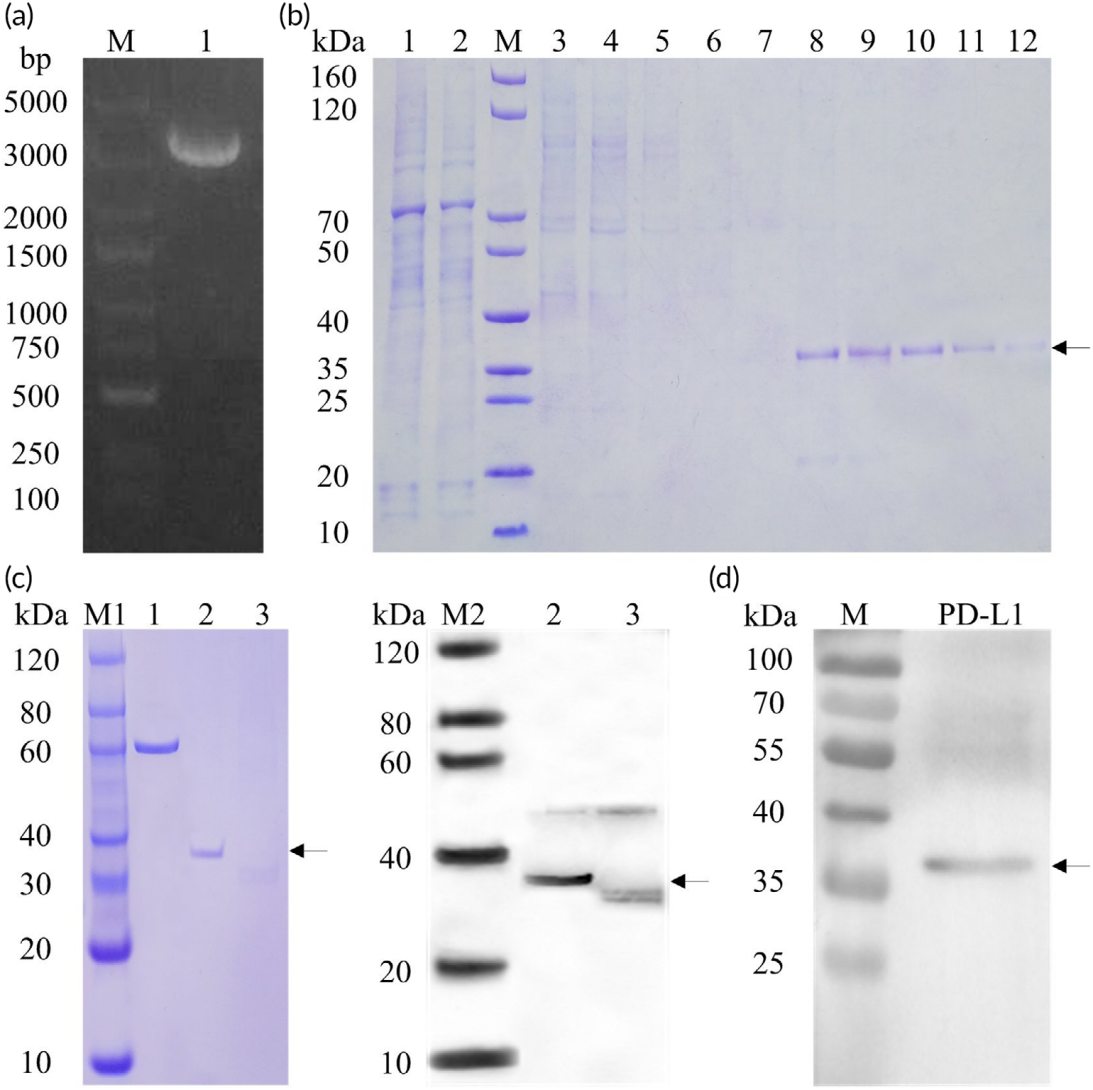
Synthesis process and identification of map‐PD‐L1. (a) Restriction digest analysis of the map‐PDL1 extraction plasmid (Lane M: DNA marker; Lane 1: transfection‐grade plasmid). (b) Ni‐IDA affinity purified map‐PD‐L1 was mainly present in the eluted fraction (Lanes 6–10), as indicated by the arrow (Lane M: SDS‐PAGE protein marker; Lane 1: supernatant after centrifugation; Lane 2: unbound sample after incubation with Ni‐IDA; Lane 3–5: 50 mM Imidazole elution fraction; Lane 6–10: 100 mM imidazole elution fraction; Lane 11–12: 500 mM imidazole elution fraction). (c) Identification of purified map‐PD‐L1 (Lane 1: BSA [1.0 μg]; Lane 2: map‐PD‐L1 protein [0.4 μg] [reduced]; Lane 3: map‐PD‐L1 protein [0.4 μg] [nonreduced]; M1: SDS‐PAGE Marker; M2: Western Blot Marker [anti‐His tag antibody]); (d) Identification of purified map‐PD‐L1 (anti‐PD‐L1 antibody, NOVUS, NBP1‐76769). map‐PD‐L1, membrane‐anchored‐protein PD‐L1

The study design is as shown in Figure 1, in order to verify whether the extracellular domain of PD‐L1 can be anchored and expressed in donor organs, and play a role in regulating immune response and preventing acute rejection. We used DNA sequencing to identify the expression vector pcDNA3.1(−) containing the PD‐L1 + APT542 gene. Once the correct gene was obtained, a transfection‐grade plasmid was generated and screened (Figure 2a). The transfection plasmid was transfected into HEK293 cells for expression of the target protein, which was then purified using Ni‐IDA affinity chromatography (Figure 2b), ultimately yielding the fusion protein map‐PD‐L1, at >90% purity. The protein amino acid sequence of map‐PD‐L is shown in Table S1. Western blot analysis was performed to confirm that the synthesized fusion protein was recognized by anti‐His and anti‐PD‐L1 specific antibodies (Figure 2c,d).

### 3.2 Map‐PD‐L1 can be anchored to the surface of cell

**FIGURE 3:**
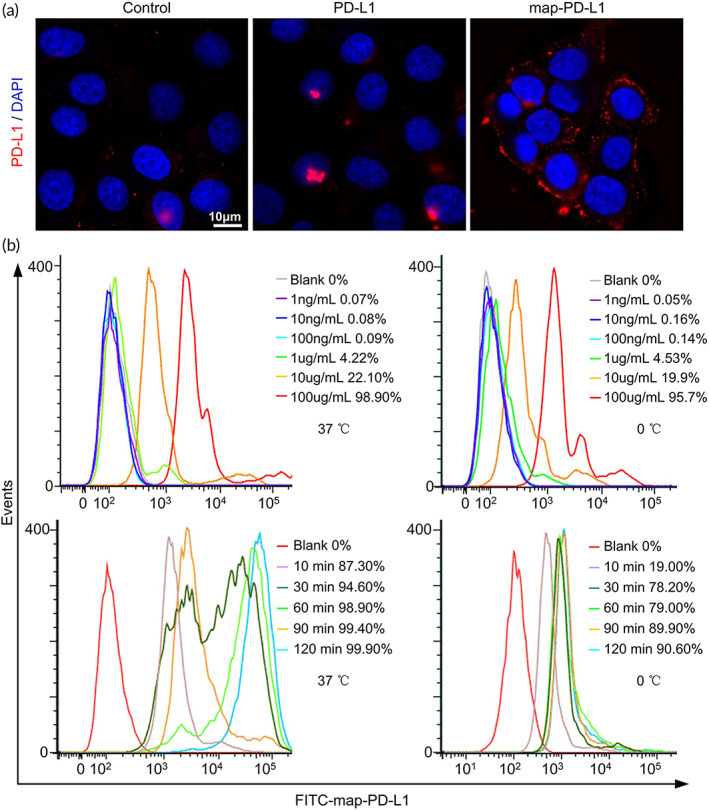
In vitro experiments to verify the anchoring functions of map‐PD‐L1. (a) Ability of map‐PD‐L1 to bind to rgEC, as analyzed by confocal laser scanning microscope. (b) Flow cytometry analysis of factors affecting map‐PD‐L1 binding to rgEC, including map‐PD‐L1 concentration, incubation time, and temperature. map‐PD‐L1, membrane‐anchored‐protein PD‐L1; rgEC, rat glomerular endothelial cell

Experimental rgEC were incubated with 5 μg/ml map‐PD‐L1 or PD‐L1, and the control group was treated with an equal volume of medium. Confocal laser scanning microscope analysis revealed that map‐PD‐L1 could be anchored on the cell membrane of rgEC (Figure 3a). Furthermore, we found that the anchoring efficiency of map‐PD‐L1 to rgEC was proportionate to the concentration of map‐PD‐L1. When incubated at 37°C for 1 h, the anchoring efficiency of 100 μg/ml map‐PD‐L1 reached 100%. Similarly, incubation at 4°C for 1 h with 100 μg/ml map‐PD‐L1 led to an anchoring efficiency greater than 95%. The anchoring efficiency at different incubation times was measured using 50 μg/ml map‐PD‐L1. Our results revealed that the anchoring efficiency of map‐PD‐L1 was proportionate to the incubation time. The anchoring efficiency was almost 90% after 10 min at 37°C but reached 100% after a 1‐h incubation. The anchoring efficiency after a 30‐min incubation at 0°C approached 80% (Figure 3b).

### 3.3 Map‐PD‐L1 can bind to PD‐1 and promote T cell apoptosis and inhibits T cell activation

A sandwich ELISA was used to detect the binding between the recombinant map‐PD‐L1 and PD‐1 (schematic diagram of ELISA was shown in Figure S1A). Through the standard ELISA procedure and statistical analysis of data, we found that the map‐PD‐L1 with anchoring function could be detected by ELISA to bind to PD‐1, while the free His‐tagged PD‐L1 failed to anchor to the endothelial cell surface, and the content of PD‐1 could hardly be detected. This result confirmed that map‐PD‐L1 could bind to PD‐1, and the binding efficiency was proportional to the incubation concentration of PD‐1 (Figure S1B).

**FIGURE 4:**
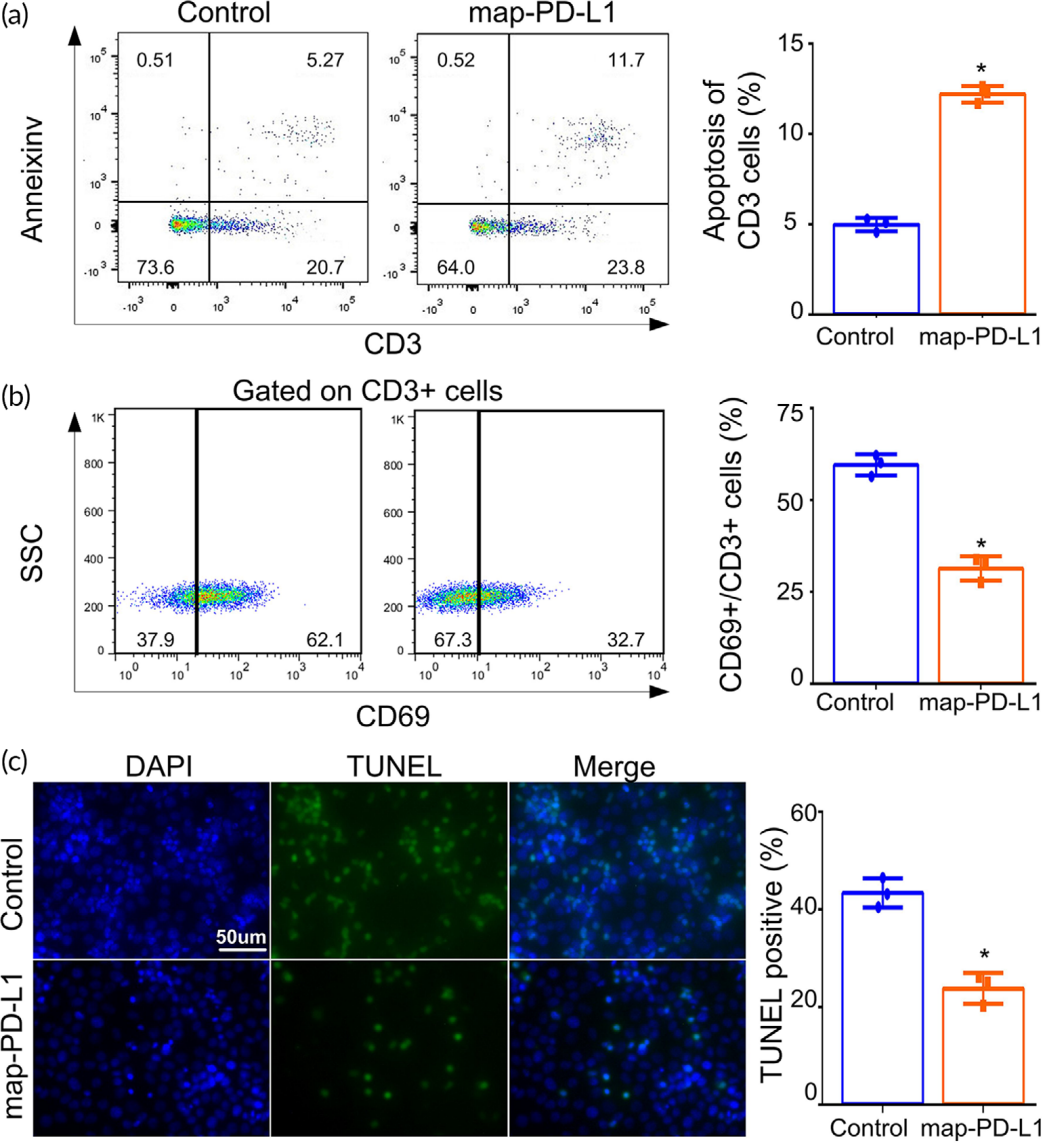
In vitro experiments to verify the biological functions of map‐PD‐L1. Spleen lymphocytes were cocultured with rgEC. For the T cell activation assay, cells were cocultured with anti‐CD3 and anti‐CD28 antibodies for 1 day as a stimulation protocol. In the experimental group, map‐PD‐L1 was anchored to rgEC. Apoptosis (a) and activation (b) of T cells were analyzed by PI and annexin V staining, and CD69 molecular markers, respectively. (c) Detection of rgEC apoptosis after coculture with spleen lymphocytes using a TUNEL Apoptosis Assay Kit. Original magnification, ×400. map‐PD‐L1, membrane‐anchored‐protein PD‐L1; rgEC, rat glomerular endothelial cell

The mixed culture of rgEC and rat spleen lymphocytes confirmed that map‐PD‐L1 anchored on the cell membrane of rgEC could promote T cell apoptosis. The rate of T cell apoptosis in the anchored map‐PD‐L1 group was much higher than that in control group (11.7% vs. 5.27%, *p* < 0.05) (Figure 4a). Map‐PD‐L1 also inhibited the activation of T cells. The proportions of CD69‐positive T cells in the control group and map‐PD‐L1 group were 62.1% and 32.7%, respectively, and the difference was statistically significant (Figure 4b). Further analysis showed that the apoptosis of endothelial cells in the anchored map‐PD‐L1 group was significantly improved compared to the control (Figure 4c).

### 3.4 Map‐PD‐L1 can be locally anchored in the donor kidney

**FIGURE 5:**
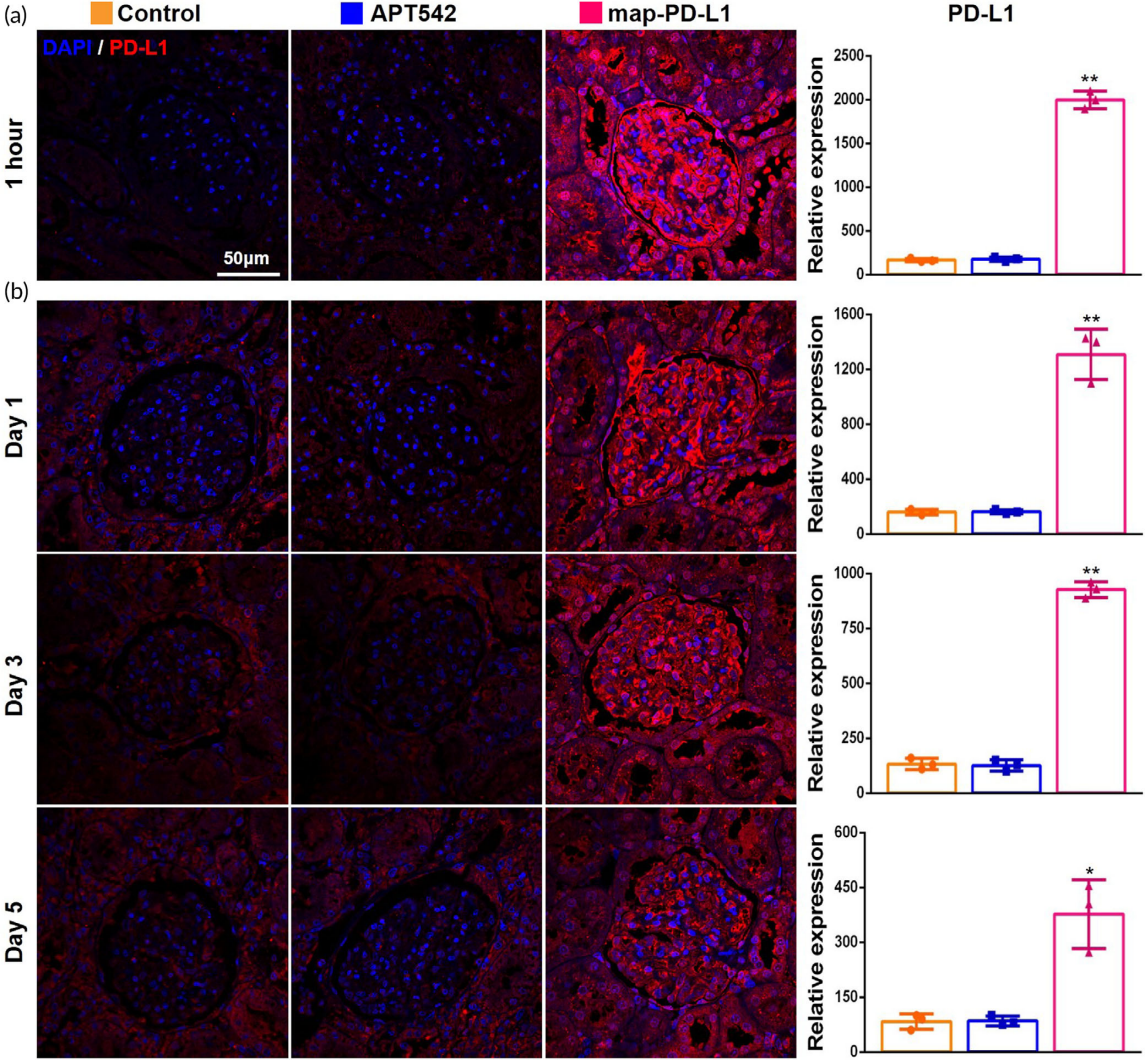
Map‐PD‐L1 can be effectively anchored in the donor kidney. (a) Representative images for the expression of PD‐L1 in allograft tissue obtained 1 h after perfusion in vitro; (b) expression of PD‐L1 in allograft lesions 1, 3, and 5 days after kidney transplantation. map‐PD‐L1, membrane‐anchored‐protein PD‐L1

We sought to confirm that map‐PD‐L1 could be effectively anchored to the donor kidney during the perfusion phase in vitro. We used immunofluorescence to detect the expression of PD‐L1 in the donor kidney, which had been perfused for 1 h prior to transplantation. We found that compared to the other two groups, PD‐L1 was highly expressed in the donor kidney of the PD‐L1‐treated group (Figure 5a). Furthermore, representative images of PD‐L1 staining showed mild overexpression 5 days after transplantation, but strong overexpression 1 and 3 days after transplantation when comparing the experimental group expressing map‐PD‐L1 to the other groups (Figure 5b).

### 3.5 Map‐PD‐L1 improves graft function and prolongs graft survival in vivo

**FIGURE 6:**
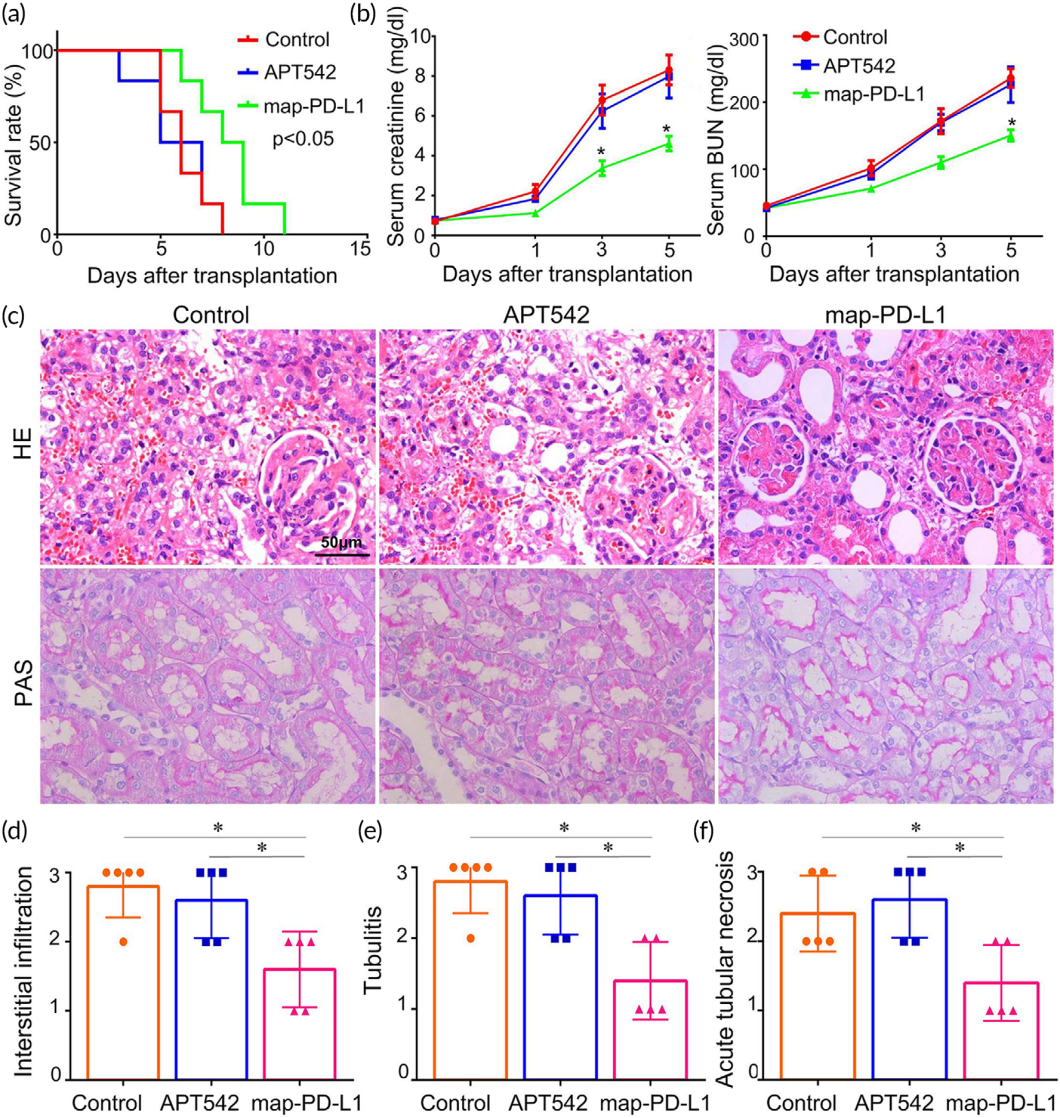
Map‐PD‐L1 alleviates pathological damage of acute renal allograft rejection and prolongs graft survival in vivo. (a) Survival of allografts from donors. Blood samples were collected prior to surgery and 1, 3, and 5 days after kidney transplantation to detect serum creatinine and BUN levels (b). Samples shown here were obtained 5 days after kidney transplantation. The graft sections were stained with H&E and PAS posttransplant (c); Histologic evaluation and classification of different groups (d–f). Histological classifications were evaluated according to the semiquantitative Banff scoring criteria: 0, absent; 1, mild; 2, moderate; 3, prominent (*p < 0.05). BUN, blood urea nitrogen; H&E, hematoxylin and eosin; map‐PD‐L1, membrane‐anchored‐protein PD‐L1; PAS, Periodic acid–Schiff

By constructing a rat kidney transplantation model, we confirmed that the survival time of the anchored map‐PD‐L1 group was significantly longer than that of both the APT542 and control groups (*p* < 0.05) (Figure 6a). The serum creatinine and urea nitrogen levels in the map‐PD‐L1 group decreased compared with the other groups. Furthermore, significant differences were apparent in the serum creatinine level at 3 and 5 days postsurgery (*p* < 0.05) (Figure 6b). There was also a significant difference in BUN serum levels 5 days after surgery (*p* < 0.05) (Figure 6b).

### 3.6 Map‐PD‐L1 alleviates pathological damage of acute renal allograft rejection

We used a kidney transplant model with a high degree of rejection after transplantation, with BN rats as donors and Lewis rats as recipients. The kidney specimens were obtained 5 days after operation. Using H&E and PAS staining (Figure 6c), we observed differences between the experimental and control groups in the degree of renal interstitial infiltration (Figure 6d), tubulitis (Figure 6e), and acute tubular necrosis (Figure 6f).

### 3.7 Map‐PD‐L1 reduces the infiltration of inflammatory cells in the allograft

**FIGURE 7:**
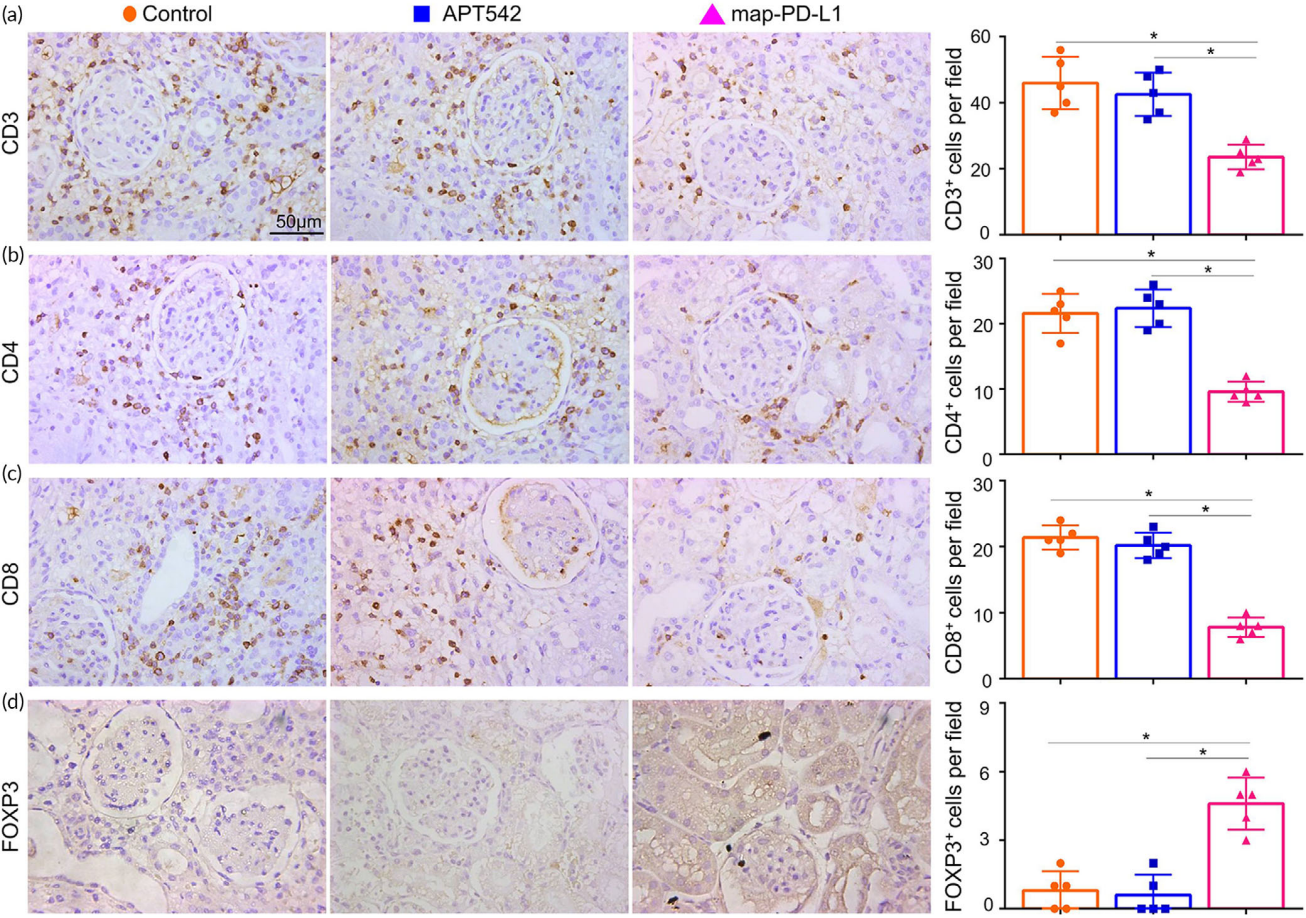
Effect of map‐PD‐L1 on immune T cells infiltration of the allograft. Samples shown here were obtained 5 days after kidney transplantation. Representative images and quantitative cell count of CD3+ (a), CD4+ (b), CD8+ (c), and FOXP3+ (d) T cells infiltrating allograft lesions. Data are presented as mean ± SD of five random views per sample (*p < 0.05). map‐PD‐L1, membrane‐anchored‐protein PD‐L1

Acute rejection is mainly mediated by T cells, and increased local infiltration of T cells is a visual manifestation of acute rejection. Using kidney specimens obtained 5 days after transplantation, we observed significantly reduced infiltration of CD3+ (Figure 7a), CD4+ (Figure 7b), CD8+ cells (Figure 7c), as well as CD68+ cells (Figure S2), in the group expressing map‐PD‐L1 (*p* < 0.05). Furthermore, using RT‐PCR we found that the expression levels of inflammatory factors, including IFN‐γ, TNF‐α, IL‐2, IL‐4, IL‐6, and IL‐17, were decreased in the map‐PD‐L1 group compared with the other groups (Figure S3A) (*p* < 0.05).

### 3.8 Map‐PD‐L1 increases the infiltration of Treg cells in the allograft

Since the presence of Tregs can be beneficial for graft outcome, we investigated the effect of map‐PD‐L1 on recipient Treg cells. Kidney specimens were obtained 5 days after transplantation and RT‐PCR was used to analyze *Foxp3* mRNA levels, as Foxp3 is a marker of Tregs. We found an increase in *Foxp3* mRNA levels in the map‐PD‐L1 group compared to the control groups (Figure S3B). Foxp3+ Treg cells were visible in allograft of the group expressing anchored map‐PD‐L1, whereas in contrast, few Foxp3+ cells were observed in the control group (*p* < 0.05) (Figure 7d).

### 3.9 Safety evaluation of map‐PD‐L1

**FIGURE 8:**
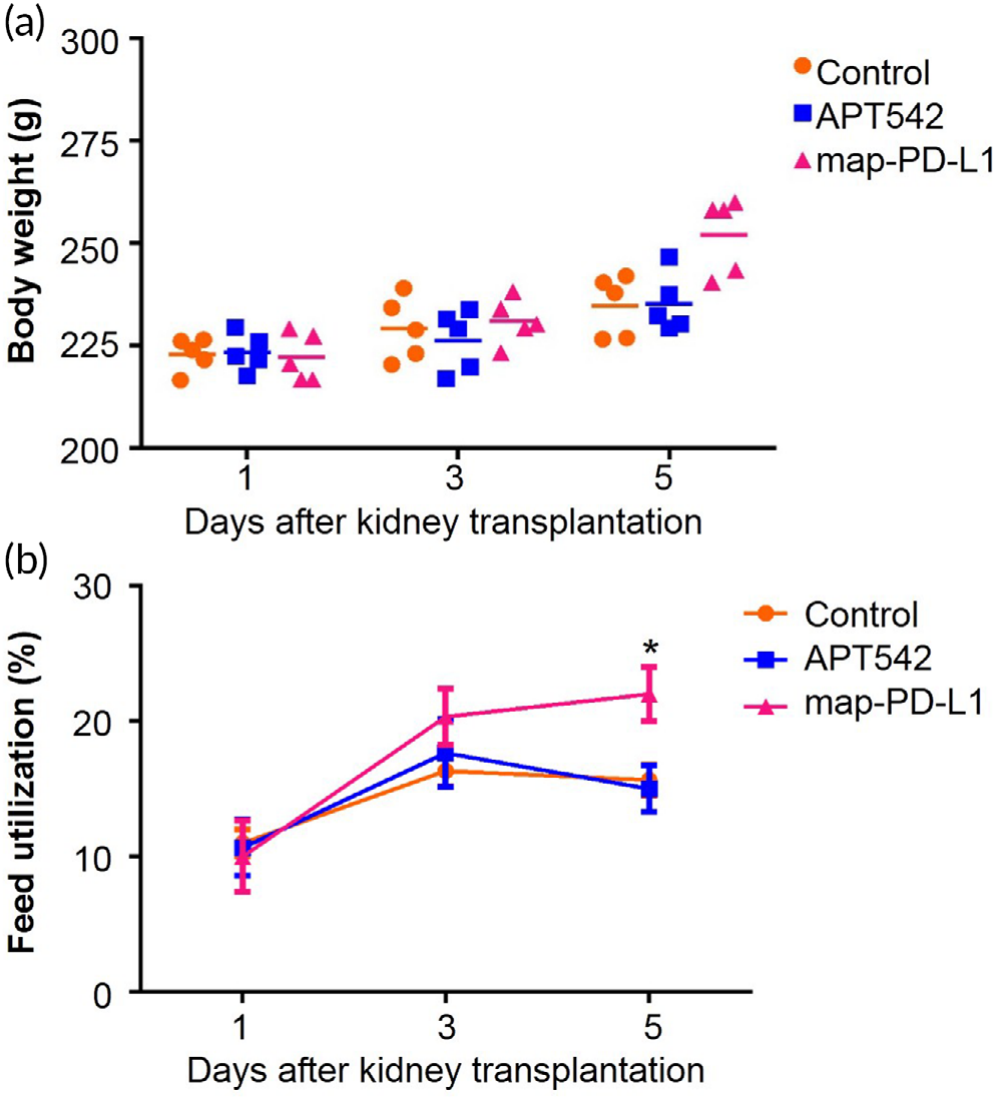
Body weight and feed utilization in transplant recipients. (a) Average body weight of control, APT542 and map‐PD‐L1 groups. (b) Feed utilization of the control, APT542 and map‐PD‐L1 groups. Data are expressed as mean ± SD. *p < 0.05, as determined using a Student's *t*‐test. map‐PD‐L1, membrane‐anchored‐protein PD‐L1

To explore whether map‐PD‐L1 could be released into the blood circulation quickly after the kidney was reopened to the blood supply, causing the circulating level of PD‐L1 to increase and causing systemic effects, we collected serum samples at 1, 3, and 5 days postsurgery, and used ELISA to detect the expression level of PD‐L1. However, the level of PD‐L1 was blow the lowest standard and the level of confident detection of the ELISA (data not shown). In addition, the mean body weight and feed utilization of the recipients are shown in Figure 8a,b, respectively. We found that the body weight of map‐PD‐L1‐treated rats was significantly different from that of the control and APT542 groups on the fifth day after transplantation (*p* < 0.05), and there were significant differences in food utilization between the groups (*p* < 0.05).

## 4 DISCUSSION

Localized treatment to the organ of interest prior to transplantation could effectively reduce the risk of systemic side effects.^19^ Endothelial cells of donor organs are the first line of defense against recipient immune cells, and the interaction between recipient lymphocytes and donor endothelial cells is critical in initiating immune‐mediated rejection.^20^, ^21^ Therefore, in vitro perfusion could be useful in defending against recipient immune cells attack on donor endothelial cells, and this strategy could provide a new immune induction scheme after organ transplantation.

With the development of protein anchoring technology, researchers have begun to use anchors to express target proteins on the cell surface in an “implantable” way.^22^, ^23^ Studies have confirmed that anchoring of the extracellular domain of the target protein on the surface of the target cell, using structures such as APT542, results in increased activity compared to the free extracellular domain.^24^, ^25^ In this study, we found that map‐PD‐L1 could anchor to the surface of rgECs in a fast and efficient manner. In addition, our in vitro splenic lymphocyte coculture assay confirmed that map‐PD‐L1 could induce T cell apoptosis and inhibit T cell activation. Furthermore, we found that after anchoring map‐PD‐L1 on the surface of rgEC, they became resistant to the cell death effect mediated by mixed lymphocyte culture. We confirmed, for the first time, that the extracellular domain of PD‐L1 retains its biological function after being anchored to the cell surface.

Anchoring technology protects target proteins from being shed from the cell surface, thus prolonging their half‐life.^26^, ^27^ The half‐life of PD‐L1 was about 18 h,^28^ but we found that the experimental anchor group still had higher expression of PD‐L1 than the other two groups on the fifth day after transplantation. This suggests that we were able to significantly prolong the half‐life of PD‐L1 by anchoring it to the cell surface. Although the local residence time of PD‐L1 in the graft was only about 5 days, it is enough to reduce the incidence of acute rejection as the earliest several days after transplantation have the highest risk for dramatic rejection. This is why most induction therapies with high doses of immunosuppressive drugs were administered only within 3 days after surgery.^29^ Therefore, we believe that the anchoring of map‐PD‐L1 in vitro holds promise as a feasible induction therapy.

In addition, we also determined that low temperature did not affect the anchoring function of map‐PD‐L, as anchoring remained efficient during cold perfusion. Although normothermic machine perfusion had been applied in clinical practice, static cold storage was still the main method of preservation.^30^, ^31^ We found that the anchoring of map‐PD‐L1 was easier to perform in a static storage environment. Furthermore, this method did not increase the cost, and the operation was simple, indicating that this strategy could have a large clinical value.

Recent studies suggest that high expression of PD‐L1 is the key to avoiding an immune attack and achieving immune tolerance after transplantation.^32^, ^33^ Therefore, many studies have focused on inducing high expression of PD‐L1.^34^, ^35^ However, there is still no effective way to upregulate the local PD‐L1 expression in target organs. In our study, by perfusing the donor kidney with map‐PD‐L1 prior to transplantation, we were able to induce high levels of expression of PD‐L1 in a targeted manner. We found that the experimental group expressing anchored map‐PD‐L1 displayed improved graft function and survival time, suggesting that map‐PD‐L1 could be anchored on the surface of donor renal endothelial cells, and perform the biological function of PD‐L1 in the recipient. Through histopathological analysis, we found that map‐PD‐L1 can effectively alleviate an acute rejection reaction in the early stage after transplantation.

The mechanism of how PD‐L1 affects T cells is clear. Numerous studies have confirmed that the interaction of PD‐1 with PD‐L1 could effectively drive T cell dysfunction. In this study, we found map‐PD‐L1 could promote T cell apoptosis and inhibit T cell activation, thereby reducing the infiltration of local T cells in the graft. Interestingly, we also found macrophage infiltration in the experimental group was significantly lower than that of the other groups. Moreover, the expression levels of macrophage‐related inflammatory cytokines, such as TNF‐α and IL‐6, were also significantly downregulated, suggesting that, consistent with published research,^36^ local high expression of PD‐L1 also had an effect on macrophages. More importantly, we found that Foxp3+ cells appeared in the experimental group, but were hardly seen in the two control groups. Thus, after map‐PD‐L1 modification, T cells could be induced to differentiate into Treg cells, so as to further regulate the local immune response of the graft. Tregs have been shown to prolong allograft survival indefinitely without long‐term systemic immunosuppression, and play a critical role in transplantation tolerance.^37^, ^38^ Some studies have illustrated the role of PD‐1/PD‐L1 axis in Treg development and function.^39^ In our study, we further confirmed that, after anchoring on to the cell surface, the extracellular domain of PD‐L1 could also regulate the differentiation of Treg cells, which might play a long‐term role in inducing local immune tolerance. We also explored the effect of map‐PD‐L1 on humoral immunity, but the data were not specifically shown in the study because the differences between groups were not obvious, which indicated that map‐PD‐L1 worked by affecting T cells rather than B cells.

It should also be noted that our study was not the first to propose the protective effect of PD‐L1 in the kidney. In Jaworska's reasech,^40^ they found that both antibody neutralization and genetic deficiency of PD‐L1 could accelerate kidney damage during ischemia reperfusion injury. However, the study did not explore the protective effect on the kidney from the aspect of overexpression of PD‐L1 in the kidney. We cleverly used the stage of donor kidney in vitro and achieved rapid upregulation of PD‐L1 expression in donor kidney through cell anchoring technology, further confirming the protective effect of PD‐L1 on the donor kidney in the rat kidney transplantation model. Although the local expression of map‐PD‐L1 in the donor kidney only lasts for 5 days, the immune induction therapy after kidney transplantation is mostly completed within 3–5 days in clinic. More importantly, without the use of any immunosuppressive drugs, map‐PD‐L1 prolonged the survival time of the graft by nearly double. These clearly indicate that more efforts are needed to extend the expression time of map‐PD‐L1 in the donor kidney and promote its clinical transformation.

Preliminary safety analysis showed that map‐PD‐L1 did not increase the circulating concentration in vivo. We detected the circulating level of PD‐L1 in each group at different time points, but the PD‐L1 level did not achieve the lower limits of detection in the ELISA assay, suggesting that map‐PD‐L1 would not abruptly release from the donor kidney into the blood, which could cause some unpredictable side effects. We further analyzed the body weight and feed consumption in the recipient, and concluded that map‐PD‐L1 did not induce toxic side effects in recipients. These results provide a preliminary safety analysis for the further clinical transformation of map‐PD‐L1.

## 5 CONCLUSION

In summary, we have successfully constructed a novel fusion protein map‐PD‐L1, which can successfully anchor on the organ endothelial cell and efficiently reduce acute rejection without any immunosuppressant. The anchoring property has extended the half‐life of the PD‐L1 in vivo from 18 h to about 5 days, which exactly covers the highest risk period for allograft rejection. The induction of in situ Treg suggests this regimen may have a long‐term effect in protecting the allograft function. Moreover, the easy‐handling property makes this regimen easy for translation in clinical practice. Therefore, the ex vivo perfusion of donor organ with map‐PD‐L1 might provide a new strategy for organ‐targeted induction therapy in transplantation.

## AUTHOR CONTRIBUTIONS

**Zihuan Luo:** Conceptualization (lead); data curation (lead); methodology (equal); writing – original draft (lead). **Tao Liao:** Data curation (equal); methodology (lead); writing – original draft (equal). **Yannan Zhang:** Resources (equal); software (lead); writing – review and editing (equal). **Haofeng Zheng:** Methodology (equal); validation (equal). **Qipeng Sun:** Methodology (equal); validation (equal). **Fei Han:** Methodology (equal); resources (equal); validation (equal). **Maolin Ma:** Formal analysis (equal). **Yongrong Ye:** Formal analysis (equal); methodology (equal). **Qiquan Sun:** Conceptualization (equal); funding acquisition (lead); writing – review and editing (lead).

## CONFLICTS OF INTEREST

The authors declare no conflicts of interest.

### PEER REVIEW

The peer review history for this article is available at https://publons.com/publon/10.1002/btm2.10316.

## Supporting information


**Table S1** The protein amino acid sequence of map‐PD‐L
**Table S2** Primers for RT‐PCR
**Figure S1**. map‐PD‐L1 can bind to PD‐1
**(A)** Schematic diagram of ELISA. **(B)** map‐PD‐L1 could bind to PD‐1, and the binding efficiency was proportional to the incubation concentration of PD‐1.
**Figure S2**. Effect of map‐PD‐L1 inhibition on macrophage cells infiltrating renal graft
Specimens used in this analysis were obtained 5 days after kidney transplantation. Representative images and quantification of CD68 cell infiltration in allograft lesions. Quantitative cell counts of CD68+ cells. Results represent mean cell numbers ± SD of 5 random views per sample (*P < 0.05).
**Figure S3**. map‐PD‐L1 preconditioning of the donor kidney regulated the level of graft inflammatory factors after transplantation
Using RT‐PCR, the levels of *IFN‐γ*, *TNF‐α*, *IL‐2*, *IL‐4*, *IL‐6* and *IL‐17* in the graft were all found to be lower in the map‐PD‐L1 group (**A**), while, the levels of *FOXP3* was higher in the map‐PD‐L1 group (**B**). Data is shown as mean ± SEM, and groups consisted of at least 5 animals. * P < 0.05, when compared to the control group using a Student's *t*‐test.

## Data Availability

The data that support the findings of this study are available from the corresponding author upon reasonable request.
