# Supplementary material for: Ex vivo anchored PD‐L1 functionally prevent in vivo renal allograft rejection
Source: Bioeng Transl Med. 2022 Apr 6;7(3):e10316. doi: 10.1002/btm2.10316 (PMC9472007; doi:10.1002/btm2.10316)
Supplement: Supplementary file 1 — Table S1 The protein amino acid sequence of map‐PD‐L Table S2 Primers for RT‐PCR Figure S1. map‐PD‐L1 can bind to PD‐1 (A) Schematic diagram of ELISA. (B) map‐PD‐L1 could bind to PD‐1, and the binding efficiency was proportional to the incubation concentration of PD‐1. Figure S2. Effect of map‐PD‐L1 inhibition on macrophage cells infiltrating renal graft Specimens used in this analysis were obtained 5 days after kidney transplantation. Representative images and quantification of CD68 cell infiltration in allograft lesions. Quantitative cell counts of CD68+ cells. Results represent mean cell numbers ± SD of 5 random views per sample (*P < 0.05). Figure S3. map‐PD‐L1 preconditioning of the donor kidney regulated the level of graft inflammatory factors after transplantation Using RT‐PCR, the levels of IFN‐γ, TNF‐α, IL‐2, IL‐4, IL‐6 and IL‐17 in the graft were all found to be lower in the map‐PD‐L1 group (A), while, the levels of FOXP3 was higher in the map‐PD‐L1 group (B). Data is shown as mean ± SEM, and groups consisted of at least 5 animals. * P < 0.05, when compared to the control group using a Student's t‐test. [file BTM2-7-e10316-s001.docx]

**Supplementary Materials**

**Supplementary Table 1 The protein amino acid sequence of map-PD-L**

| map-PD-L1 Protein Length=245 MW=27616.3 pI=7.62 |
| --- |
| 1 MHHHHHHAFT ITAPKDLYVV EYGSNVTMEC RFPVEQKLDL LALVVYWEKE DKEVIQFVEG  61 EEDLKPQHSS FRGRAFLPKD QLLKGNAVLQ ITDVKLQDAG VYCCMISYGG ADYKRITLKV  121 NAPYRKINQR ISMDPATSEH ELMCQAEGYP EAEVIWTNSD HQSLSGETTV TTSQTEEKLL  181 NVTSVLRVNA TANDVFHCTF WRVHSGENHT AELIIPELPV PRLPHNRTHG SSKSPSKKKK  241 KKPGDC |

**Supplementary Table 2 Primers for RT-PCR**

| Gene | Forward primer | Reverse primer |
| --- | --- | --- |
| FOXP3 | GTATCACTTGCCACTAACCC | TTGTCCAGCTTGACCACA |
| IFN-γ | GCTAGATTCTGGTGACAGCTGGTG | CACCAGCTGTCACCAGAATCTAGC |
| TNF-α | AAATGGGCTCCCTCTCATCAGTTC | TCTGCTTGGTGGTTTGCTACGAC |
| IL-2 | GCAGGCCACAGAATTGAAAC | CCAGCGTCTTCCAAGTGAA |
| IL-4 | GTACCAGACGTCCTTACGGC | CAGACCGCTGACACCTCTAC |
| IL-6 | CTTCCAGCCAGTTGCCTTCT | GACAGCATTGGAAGTTGGGG |
| IL-17 | ACATGTAAGGCAGCGGTACT | GCTCAGAGTCCAGGGTGAAG |
| GAPDH | CATCAACGACCCCTTCATTGAC | ACTCCACGACATACTCAGCACC |

**Supplementary Figure and Figure Legend:**

**
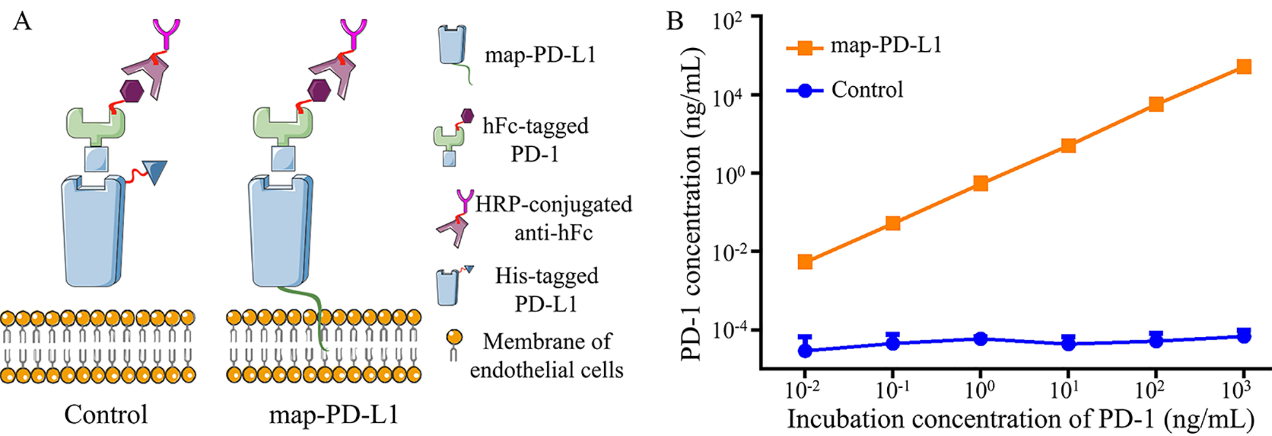
**

**Supplementary Figure 1.** **map-PD-L1 can bind to PD-1**

**(A)** Schematic diagram of ELISA. **(B)** map-PD-L1 could bind to PD-1, and the binding efficiency was proportional to the incubation concentration of PD-1.

**
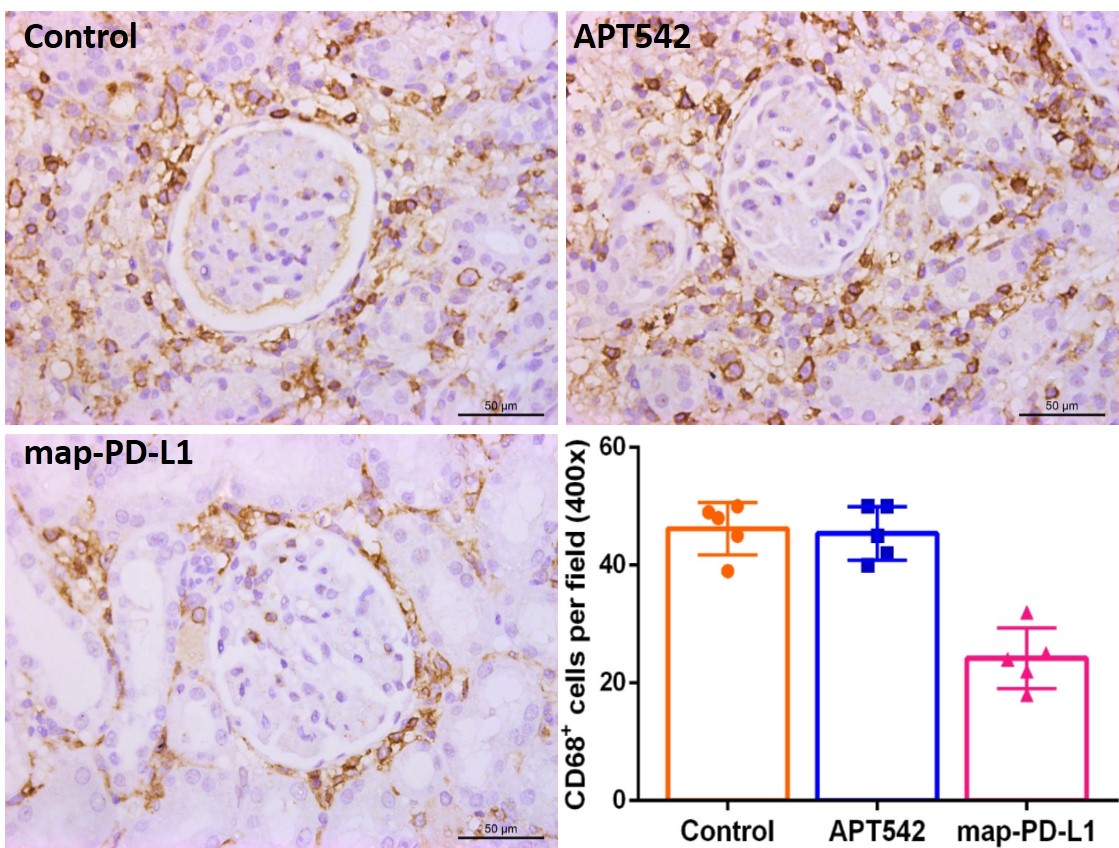
**

**Supplementary Figure 2.** **Effect of map-PD-L1 inhibition on macrophage cells infiltrating renal graft**

Specimens used in this analysis were obtained 5 days after kidney transplantation. Representative images and quantification of CD68 cell infiltration in allograft lesions. Quantitative cell counts of CD68+ cells. Results represent mean cell numbers ± SD of 5 random views per sample (*P < .05).

**
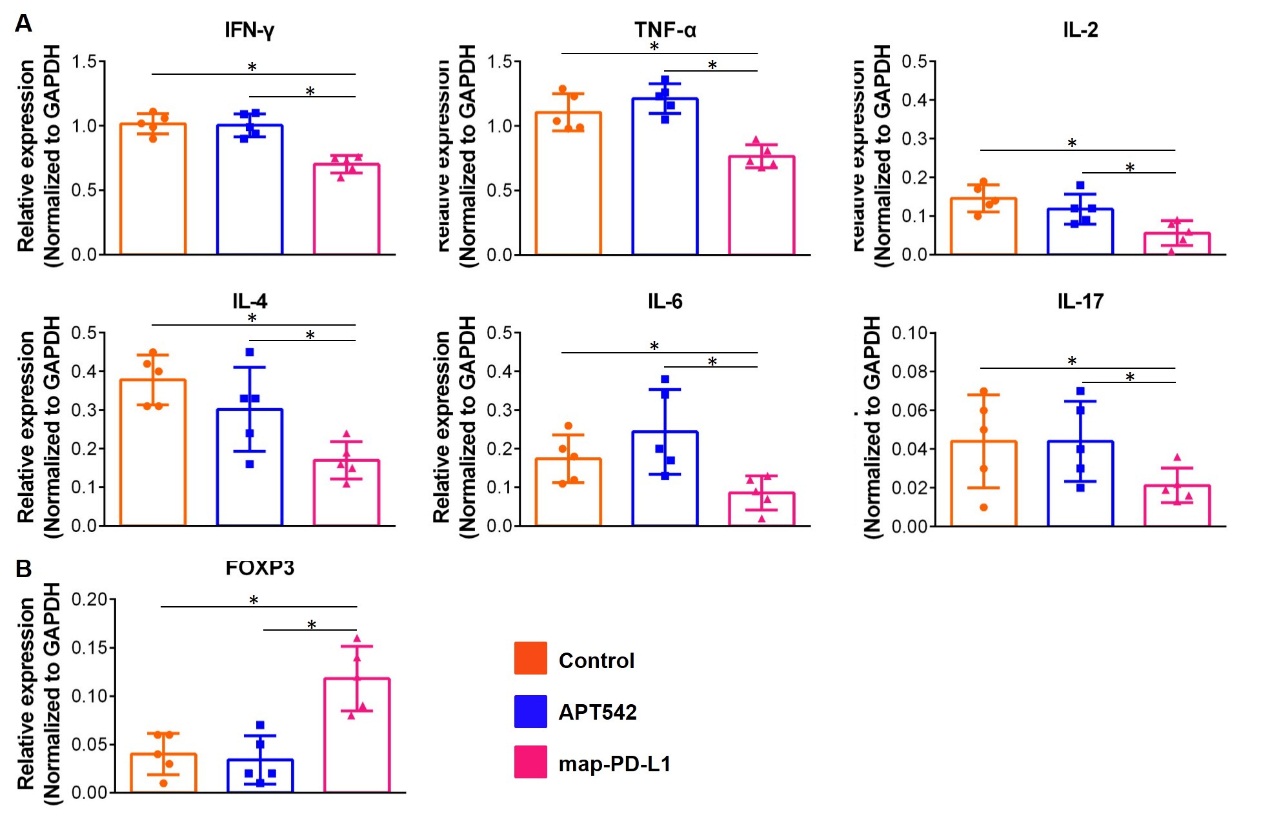
Supplementary Figure 3.** **map-PD-L1 preconditioning of the donor kidney regulated the level of graft inflammatory factors after transplantation**

Using RT-PCR, the levels of *IFN-γ, TNF-α, IL-2, IL-4, IL-6* and *IL-17* in the graft were all found to be lower in the map-PD-L1 group (**A**), while, the levels of *FOXP3* was higher in the map-PD-L1 group (**B**). Data is shown as mean ± SEM, and groups consisted of at least 5 animals. * P < 0.05, when compared to the control group using a Student’s *t*-test.
